# Group-based music intervention in Parkinson’s disease – findings from a mixed-methods study

**DOI:** 10.1177/0269215520907669

**Published:** 2020-02-19

**Authors:** Petra Pohl, Ewa Wressle, Fredrik Lundin, Paul Enthoven, Nil Dizdar

**Affiliations:** 1 Department of Activity and Health, and Department of Health, Medicine, and Caring Sciences, Linköping University, Linköping, Sweden; 2 Department of Acute Internal Medicine and Geriatrics, Department of Social and Welfare Studies, Linköping University, Linköping, Sweden; 3 Department of Neurology, and Department of Clinical and Experimental Medicine, Linköping University, Linköping, Sweden; 4 Pain and Rehabilitation Centre, and Department of Health, Medicine, and Caring Sciences, Linköping University, Linköping, Sweden

**Keywords:** Parkinson’s disease, randomized controlled trial, mixed-methods, music-based intervention, rehabilitation

## Abstract

**Objective:**

To evaluate a group-based music intervention in patients with Parkinson’s disease.

**Design:**

Parallel group randomized controlled trial with qualitative triangulation.

**Setting:**

Neurorehabilitation in primary care.

**Subjects:**

Forty-six patients with Parkinson’s disease were randomized into intervention group (*n* = 26), which received training with the music-based intervention, and control group (*n* = 20) without training.

**Interventions:**

The intervention was delivered twice weekly for 12 weeks.

**Main measures:**

Primary outcome was Timed-Up-and-Go subtracting serial 7’s (dual-task ability). Secondary outcomes were cognition, balance, concerns about falling, freezing of gait, and quality of life. All outcomes were evaluated at baseline, post-intervention, and three months post-intervention. Focus groups and individual interviews were conducted with the intervention group and with the delivering physiotherapists.

**Results:**

No between-group differences were observed for dual-task ability. Between-group differences were observed for Falls Efficacy Scale (mean difference (MD) = 6.5 points; 95% confidence interval (CI) = 3.0 to 10.0, *P* = 0.001) and for Parkinson Disease Questionnaire-39 items (MD = 8.3; 95% CI = 2.7 to 13.8, *P* = 0.005) when compared to the control group post-intervention, but these were not maintained at three months post-intervention. Three themes were derived from the interviews: *Expectations versus Results, Perspectives on Treatment Contents*, and *Key Factors for Success*.

**Conclusion:**

Patient-reported outcomes and interviews suggest that the group-based music intervention adds value to mood, alertness, and quality of life in patients with Parkinson’s disease. The study does not support the efficacy in producing immediate or lasting gains in dual-tasking, cognition, balance, or freezing of gait.

## Introduction

Music-based interventions have been suggested as adjunct management options for patients with Parkinson’s disease.^1^ Dancing, for example, has been shown to improve cognitive dual-tasking, gait-related outcomes, and global cognition.^2^ Impaired motor-cognitive dual-tasking is a common deficit in patients with Parkinson’s disease,^3^ which may be improved with targeted interventions.^4^ To increase the attractiveness, musical elements may be incorporated within such interventions,^5^ and the social benefits may be further enhanced if the intervention is group-based.^2^

The music-based intervention Ronnie Gardiner Method involves multitasking activities that require the participants to quickly shift attention between motor and cognitive tasks by interpreting visual symbols, synchronizing arms and legs in complex coordinated movements, while simultaneously pronouncing a certain word to the beat of music.^6^ Apart from multitasking, the training has the potential to improve bradykinesia, balance, freezing of gait, and cognitive function in patients with Parkinson’s disease.^6^ The therapy is practitioner-led and usually group-based for the benefits of social experiences and emotional well-being.^6^

Few studies have evaluated the music-based group therapy to date. A randomized controlled trial on stroke survivors found long-term effects on the perception of recovery, balance, grip strength, and working memory compared to controls.^7^ With respect to Parkinson’s disease, a small feasibility study revealed no between-group differences, but some tendencies towards improved mobility, cognition, and quality of life within the intervention group.^8^ A larger study is therefore needed to further investigate possible effects for patients with Parkinson’s disease.

When evaluating novel complex interventions, both objective and subjective evaluations should be considered.^9^ Including qualitative approaches provides a more in-depth understanding about the intervention. To further broaden the perspectives, the delivering professionals should be included in the evaluation.^9^ The aim of this randomized trial was to evaluate the Ronnie Gardiner Method in Parkinson’s disease and to gain insights into participants’ and therapists’ experiences of the group-based music intervention to optimize the contents, delivery, and acceptability and to facilitate further development.

## Methods

This was a single-blinded, parallel group randomized controlled trial, integrating data from qualitative methods.^10^ The study was approved by the Regional Ethical Review Board of Linköping (Dnr 2016/179-31), and all participants signed an informed consent form after receiving oral and written information. The trial was registered at ClinicalTrials.gov (NCT02999997). The study was conducted following the recommendations of Consolidating Standards for Reporting Clinical Trials (CONSORT)^11^ (Supplementary file I) with the extension to the reporting guidelines for music-based interventions^12^ (Supplementary file II) and the Consolidated Criteria for Reporting Qualitative data (COREQ).^13^

The following inclusion criteria were used for this study: community-dwelling individuals from 18 years of age with a diagnosis of Parkinson’s disease and Hoehn and Yahr^14^ up to stage 3, stable medication ⩾four months, and capacity to walk 10 m without gait assistance. To enhance the generalizability of the findings, any medical treatment, even surgical, was accepted. Neurologists screened medical records from the Departments of Neurology and Geriatrics to identify potential study participants of both genders, who were then contacted by telephone by the first author (P.P.) between December 2016 and August 2017.

Recruited patients underwent a full clinical assessment by specialists in movement disorders at the University hospital and were excluded if they had other neurological deficits or serious health conditions that would compromise participation; significant visual or hearing impairments that would make participation impossible; or severe motor fluctuations. Demographic data included age, gender, disease duration, education level, and fall history the last 12 months. In relation to fall history, patients were asked: ‘Are you experiencing poor balance?’ with a ‘Yes’ or ‘No’ response option. The Unified Parkinson’s Disease Rating Scale^15^ was also included.

After the initial examination, included patients were referred to an occupational therapist for cognitive tests, followed by physical tests by the first author. The same assessors performed cognitive and physical re-evaluations within two weeks post-intervention and three months post-intervention. Both assessors remained blind to group allocation at all evaluations.

After baseline assessments, patients were randomized into two groups: intervention group and control group. The randomization procedure was performed by an independent investigator (not part of the study) with numbers generated by a randomization website (www.random.org), and two standardized information letters were sent to the patients depending on group allocation. All patients were asked to refrain from initiating new exercise programmes or other allied health therapy interventions during the study period and were instructed not to share their treatment information to the assessors.

The primary outcome was the Timed-Up-and-Go subtracting serial-7’s measuring the effect of cognitive demands on functional mobility (motor-cognitive dual-tasking). Serial-7’s was chosen instead of the more common serial-3’s subtraction in order to place an even greater demand on the cognitive processes for attention and working memory.^16^ Secondary outcomes included (1) cognitive function (Montreal Cognitive Assessment Scale (MoCA);^17^ and three parts of the Cognitive Assessment Battery^18^ (Test Recall Test (immediate and delayed); Stroop Color-Word Test; and Symbol Digit Modalities Test)) and (2) dynamic balance (Mini-BESTest).^19^ Three questionnaires were administered (Falls Efficacy Scale International;^20^ Freezing of Gait Questionnaire;^21^ and Parkinson Disease Questionnaire 39-items Global Index Score,^22^ which rates the quality of life from excellent (zero) to very poor (100)).

Patients were tested while in on-phase, that is, within 1–2 hours after taking their anti-Parkinson medication. Due to practical reasons, it was not possible to re-test patients at the exact same time of day post-intervention. Levodopa equivalent dosage was registered before and after study completion.

Qualitative methodology was used to explore the experiences of the participants and the intervention therapists.^9^ To enhance data richness, focus group methodology was combined with individual interviews.^23^ In short, focus groups were conducted with patients from the intervention group and with the two delivering therapists by E.W. Additional face-to-face interviews were conducted with eight patients by physiotherapy students. To increase transparency and to ensure dependability and confirmability, an audit trail is provided including theoretical framework, reflexivity, and the process for qualitative analysis (Supplementary file III).

The intervention was delivered in a group setting (14 and 12 participants respectively) at a neurorehabilitation centre twice a week for 12 weeks (60 min/session). Each session was initiated with soft stretching movements and breathing exercises, followed by 50 minutes of exercises typical for the Ronnie Gardiner Method,^6^ and ended with winding down to soft classical music. Two physiotherapists, who were not authors, were engaged to provide the intervention; both were certified practitioners of the Ronnie Gardiner Method. Progression of the exercises was determined by the skill of the participants in performing the movements. Intervention details are available in Supplementary file II. A third certified practitioner who was not part of the study performed one integrity check, that is, that the protocol was followed as intended, after six weeks. Homework was given, but not on a regular basis. Training diaries were written to monitor compliance and adverse events.

The control group did not receive any competing activity but was encouraged to continue with usual care. They were offered to take part in the same intervention after the study completion.

The sample size was calculated with the Russ Lenth’s power and sample size website, based on the primary outcome Timed-Up-and-Go with a cognitive load, considering a power of 80%, a significance of 5%, and a loss rate of 20%. The calculation was inspired by the study protocol by Peters et al.,^24^ where sample size was calculated based on the cognitive task subtracting serial 3’s. A minimal clinically important difference of 3 seconds with a standard deviation of 3.6 was estimated.^24^ With these calculations, a sample size of 30 patients per group would be required to detect a difference.

Data were analysed with the SPSS software 25.0. Group differences pre-intervention were compared using Mann–Whitney *U* tests for continuous variables and chi-square tests for nominal variables. Per-protocol analysis was conducted including patients who completed assessments at pre-intervention, post-intervention, and three months post-intervention. Missing values were handled with the least square method (list wise deletion). Mixed design repeated-measures analysis of covariance (ANCOVA) was used to analyse interactions between time and group for the primary and secondary outcomes. Repeated contrast analyses were calculated post hoc between pre-intervention and post-intervention, and between pre-intervention and three months post-intervention. The influence of confounding factors was tested, and the covariates age, gender, disease duration, and cognitive function were added in the adjusted model. Pharmacological treatments were not included in the model. The assumption of sphericity was tested with Mauchly’s Test, and if not met, Greenhouse-Geisser correction was used.

Qualitative data were analysed thematically by P.P., E.W., and P.E., using qualitative content analysis^25^ (Supplementary file III). Data management and analysis were facilitated by the software Open Code 4.0 (available from Umea University at www.phmed.umu.se/enheter/epidemiologi/forskning/open-code). To increase credibility, direct quotes from the interviews are provided to support the findings.

## Results

**Figure 1.**
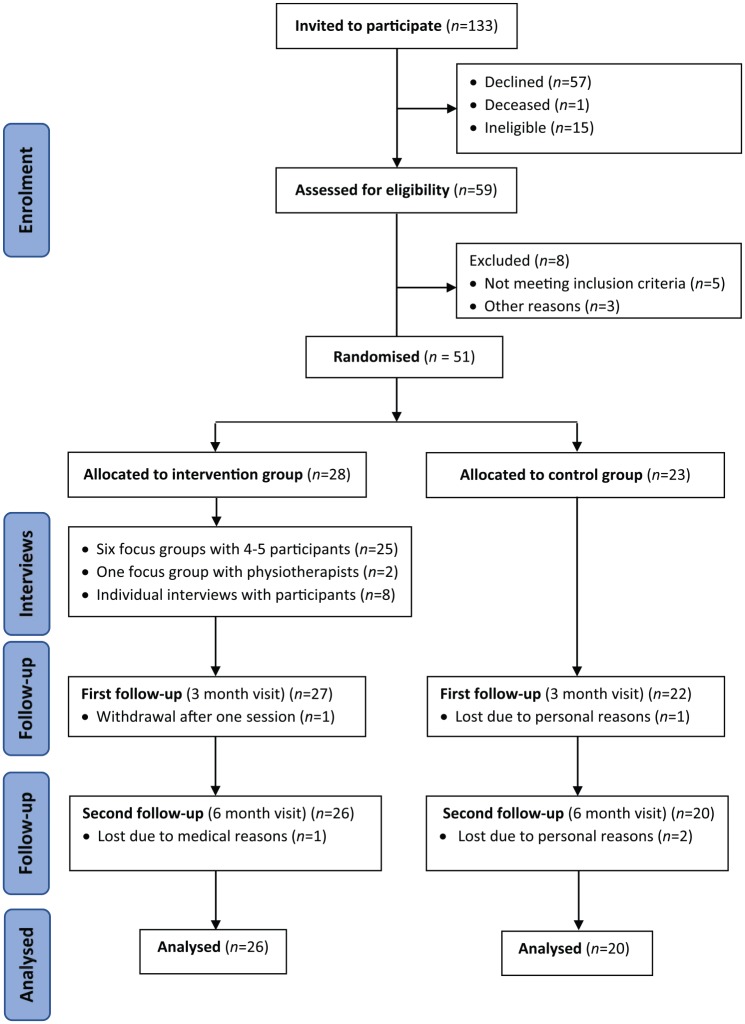
CONSORT (Schulz et al.^11^) diagram of the recruitment process adopted.

A total of 59 patients of both genders were recruited. Of these, 51 patients met the inclusion criteria and were randomized into the intervention group or the control group. Forty-six patients completed the study. Comparisons between the dropouts (*n* = 5) and non-dropouts (*n* = 46) revealed a significant gender difference as all dropouts were women. The CONSORT flowchart is presented in Figure 1.

Table 1 presents the sociodemographic and clinical characteristics of the study patients. Suggested cut-offs for MoCA^26^ indicated that 23 patients (50%) suffered from cognitive impairments (⩽25 points) at baseline. The groups were considered homogeneous at baseline. During the study, 11 (42%) intervention and 7 (35%) control group participants altered their medication. Of these, 5 (19%) intervention and 6 (30%) control group participants increased their Levodopa equivalent dosage, and 6 (23%) intervention and 1 (5%) control group participants decreased their dosage (non-significant difference between groups). Missing values (4%–17%) were due to not being able to perform tests or not fully completing questionnaires. No adverse events were reported during the intervention. Mean attendance rate was 89%, equivalent to at least 21 sessions.

**Table 1.** Sociodemographics and clinical characteristics of study participants: mean ± SD or *n* (%).

	Intervention group	Control group	*P* value
	(*N* = 26)	(*N* = 20)	
Age	69.7 ± 7.0	70.4 ± 6.0	0.649
Male	19 (73)	13 (65)	0.555
UPDRS			
Motor subscale (/108)	34.0 ± 12.9	28.6 ± 10.4	0.066
Total score (/199)	54.1 ± 17.0	47.0 ± 13.6	0.089
Hoehn & Yahr			0.293
Stage 1	3 (12)	2 (10)	
Stage 2	10 (38)	10 (50)	
Stage 3	13 (50)	8 (40)	
Education ⩾ 10 years	22 (85)	16 (80)	0.713
Disease duration, years	6.0 ± 4.4	6.8 ± 3.6	0.370
MoCA (/30)	25.5 ± 2.8	25.0 ± 3.3	0.592
Normal function (⩾26)	13 (50)	10 (50)	
Mild impairment (22–25)	10 (39)	7 (35)	
Severe impairment (⩽21)	3 (11)	3 (15)	
TUG subtracting serial-7s, s	18.5 ± 7.3	19.9 ± 9.9	0.964
Experiencing poor balance	20 (54)	17 (46)	0.711
Levodopa equivalent dosage	727.7 ± 327.3	690.0 ± 231.0	0.663

UPDRS: Unified Parkinson’s Disease Rating Scale; MoCA: Montreal cognitive assessment; TUG: Timed-Up-and-Go.

Results from the between-group comparisons for the study variables are presented in Table 2. No significant between-group difference (intervention vs. control group) was observed for the primary outcome Timed-Up-and-Go subtracting serial-7’s post-intervention and three months post-intervention. Significant between-group differences were observed at three months post-intervention for Falls Efficacy Scale International (*P* = 0.002) and for Parkinson Disease Questionnaire-39 items (*P* = 0.021) in favour of the intervention group. For the remaining variables, no significant differences were found (*P* > 0.05).

**Table 2.** Between-group comparisons of primary and secondary outcomes adjusted for multiple comparisons.

Outcome measure	Group	Estimated marginal means, mean (95% CI)			Main effects,
					*P* value
		Baseline	Post-intervention	Three months post-intervention	Time × group
Timed Up and Go subtracting 7’s (seconds) (*n* = 38; 83%)	IG (*n* = 23)	18.2 (15.5 to 20.8)	19.4 (16.0 to 22.9)	19.0 (16.3 to 21.8)	0.967
	CG (*n* = 15)	16.5 (13.2 to 19.7)	17.5 (13.2 to 21.8)	17.7 (14.2 to 21.2)	
	Contrasts		−0.2 (–5.3 to 4.9)^a^	0.4 (–4.6 to 5.3)^b^	
Montreal Cognitive Assessment scale (0–30 p) (*n* = 46; 100%)	IG (*n* = 26)	25.5 (24.8 to 26.2)	25.7 (24.8 to 26.7)	25.4 (24.4 to 26.4)	0.347
	CG (*n* = 20)	25.0 (24.2 to 25.8)	25.8 (24.8 to 26.9)	25.8 (24.7 to 27.0)	
	Contrasts		0.6 (–0.6 to 1.8)^a^	0.9 (–0.5 to 2.3)^b^	
Text immediate recall (0–21 p) (*n* = 46; 100%)	IG (*n* = 26)	4.8 (4.0 to 5.6)	4.4 (3.4 to 5.3)	5.3 (4.3 to 6.3)	0.126
	CG (*n* = 20)	5.5 (4.6 to 6.5)	3.5 (2.4 to 4.6)	5.5 (4.3 to 6.7)	
	Contrasts		−1.6 (–3.2 to 0.0)^a^	−0.5 (–2.2 to 1.2)^b^	
Text delayed recall (0–21 p) (*n* = 46; 100%)	IG (n = 26)	5.6 (4.5 to 6.7)	5.1 (3.9 to 6.3)	5.8 (4.7 to 6.9)	0.643
	CG (n = 20)	5.7 (4.4 to 7.0	4.4 (3.0 to 5.7)	5.4 (4.1 to 6.7)	
	Contrasts		−0.8 (–2.7 to 1.1)^a^	−0.5 (–2.2 to 1.1)^b^	
Stroop Color-Word Test (seconds) (*n* = 46; 100%)	IG (*n* = 26)	35.8 (29.1 to 42.6)	35.2 (28.9 to 41.5)	33.5 (28.9 to 38.2)	0.848
	CG (*n* = 20)	31.8 (24.1 to 39.5)	33.0 (25.9 to 40.2)	31.5 (26.2 to 36.8)	
	Contrasts		1.9 (–5.3 to 9.1)^a^	2.0 (–7.5 to 11.6)^b^	
Symbol Digit Modalities Test (no), (*n* = 46; 100)	IG (*n* = 26)	30.1 (27.1 to 33.2)	29.0 (25.1 to 32.9)	31.0 (27.7 to 34.2)	0.064
	CG (*n* = 20)	29.3 (25.8 to 32.7)	32.4 (27.9 to 36.8)	31.4 (27.7 to 35.1)	
	Contrasts		4.3 (0.1 to 8.4)^a^	1.3 (–1.1 to 3.8)^b^	
Mini-BESTest (0–28 p) (*n* = 44; 96%)	IG (*n* = 25)	19.4 (17.4 to 21.4)	18.8 (16.7 to 20.9)	18.6 (16.7 to 20.4)	0.964
	CG (*n* = 19)	18.9 (16.6 to 21.2)	18.6 (16.2 to 21.0)	18.3 (16.1 to 20.5)	
	Contrasts		0.2 (–1.6 to 2.1)^a^	0.2 (–1.7 to 2.2)^b^	
Falls Efficacy Scale International (16–64 p), (*n* = 40; 87%)	IG (*n* = 23)	27.0 (24.3 to 29.6)	23.8 (21.0 to 26.6)	26.6 (23.9 to 29.3)	0.002
	CG (*n* = 17)	23.2 (20.1 to 26.3)	26.5 (23.2 to 29.8)	24.9 (21.8 to 28.1)	
	Contrasts		6.5 (3.0 to 10.0)^a^	2.1 (–1.4 to 5.7)^b^	
Freezing of Gait Questionnaire (0–24 p), (*n* = 41; 89%)	IG (*n* = 24)	6.0 (4.2 to 7.9)	4.8 (2.9 to 6.6)	6.8 (4.7 to 8.9)	0.121
	CG (*n* = 17)	5.2 (3.0 to 7.5)	6.5 (4.3 to 8.7)	6.3 (3.8 to 8.8)	
	Contrast		2.5 (–0.3 to 5.3)	0.3 (–2.6 to 3.3)	
Parkinson Disease Questionnaire-39 items (0%–100%) (*n* = 35; 76%)	IG (*n* = 20)	21.9 (17.6 to 26.3)	18.6 (14.4 to 22.7)	23.2 (17.4 to 29.1)	0.021
	CG (*n* = 15)	18.3 (13.2 to 23.4)	23.2 (18.4 to 28.0)	23.8 (17.0 to 30.5)	
	Contrasts		8.3 (2.7 to 13.8)^a^	4.2 (–1.4 to 9.7)^b^	

CI: confidence interval; IG: intervention group; CG: control group.

Age, gender, disease duration, and cognitive function were added as covariates in the adjusted model. Contrasts: Pairwise contrast analysis based on mean change for intervention group versus control group for (a) pre- to post-intervention (short-term) and (b) pre-intervention to three months post-intervention (long-term).

Table 2 also shows the results from the within-group comparisons for the study variables. The intervention group had a significant improvement for Falls Efficacy Scale International (*P* = 0.022) post-intervention in relation to the baseline evaluation, that is, short-term effect, while the control group had a significant deterioration post-intervention *(P* = 0.044). The intervention group also had a significant short-term effect for Parkinson Disease Questionnaire-39 items (*P* = 0.005) post-intervention. No long-term effects were seen on any of these measurements (pre-intervention to three months post-intervention).

### Patient and therapist experiences

Seven focus groups and eight face-to-face interviews were conducted. Their characteristics are presented in Supplementary file III. All but two patients accepted to participate in the focus groups: one man declined due to illness, and one man chose not to participate. Both physiotherapists accepted to participate in a separate focus group. Three themes were derived from focus groups with patients and physiotherapists; (1) Expectations versus Results (2); Perspectives on Treatment Contents; and (3) Key Factors for Success (Table 3). The following section summarizes key themes from the data; a more extensive description is found in Supplementary file III.

**Table 3.** Themes and categories derived from the focus group discussions with patients and delivering physiotherapists regarding experiences from the group-based music intervention, the Ronnie Gardiner Method.

Theme	Category
Expectations versus Results	Anticipations
	Experienced effects
Perspectives on treatment contents	The therapy itself
	Design of treatment sessions
Key factors for success	Togetherness
	Leadership competencies
	Contextual components

The first theme describes the expectations versus the results, and two categories explain the content. The category ‘Anticipations’ describes how most patients anticipated that the music would engage them, and how the therapists anticipated that the patients would get more out of it if the training was experienced as enjoyable. The category ‘Experienced effects’ highlights perceptions of training effects. Generally, the training was perceived as energizing; that the brain ‘perked up’. Patients described feelings of improved posture and dexterity with less tremor, a smoother body, improved mood and being more cheerful, and improved endurance with better ability to concentrate. The training gave transfer effects on everyday living. Lack of training effects were also reflected upon: ‘*The tests will ultimately reveal whether there were any effects*’ (Group 2). The therapists noted subtle changes such as improved mood, endurance, a better ability to stay focused, and movement timing. The patients’ symptoms fluctuated, which made it difficult to observe effects.

The second theme describes perspectives on the treatment contents, and two categories explain the content. The category ‘The therapy itself’ illuminates how patients agreed that the therapy was something out of the ordinary, but the ‘nonsense-words’ were hard to learn. The training was easygoing, positive, and fun: ‘It’s a winning concept! It’s a combination of the programme itself, the rhythm of the music, the fellowship, and the enthusiastic leaders’. ‘Yes, the programme is so much fun that you become exhilarated’. ‘I agree, there is a lot of physical activity for one hour, and this gives you endorphins, and that is the “joy substance” of the body’. ‘Yes, and there is much memory training’ (Group 5). To coordinate arms and legs and say the correct words was a real challenge. When the concentration was broken, one was lost. The therapists observed that the patients prioritized the movements, while the words were lost. The patients needed much visual support, that is, the therapist pointed at the note system. This meant that the therapists were unable to observe the participants at all times. Therefore, for safety reasons, it was experienced as advantageous to have two instructors.

The category ‘Design of treatment sessions’ shows that the patients agreed that twice weekly one-hour sessions were just right, and this opinion was shared by the therapists. Many of the exercises were performed sitting down by choice, even when the therapists gave the option to stand up. One man from the face-to-face interviews said: ‘I preferred to stand up while doing the exercises, it’s a bit like dancing, at least sway a little (laughing). If you get it to work, it’s more fun than to just sit down and “boom,” “chic,” and so on. Music affects you very much physically!’ The therapists prioritized movement quality: ‘If they are unstable, it’s better if they sit down and do the movements properly; otherwise they will fail to perform them correctly’.

The difficulty of the exercises was also reflected upon. Many patients had great difficulties in learning the complex skills, and the therapists found it difficult to negotiate the right level to fit everyone, because the group was heterogeneous. Instead they tried to compromise, but this had consequences for the patients who were quick learners: ‘I had expected the training to be much harder and more challenging, I wanted to challenge my abilities more than it did. Only at the end they increased the speed and it became more challenging to me’ (man, individual interviews). For this patient, the exercises soon became repetitive.

There were conflicting ideas about how to improve the sessions. Patients suggested adding more homework, but the therapists disagreed; in their experience, homework was rarely carried out. Patients also suggested to combine the therapy with other therapy-specific activities, because ‘this is not enough as physical exercise’, and ‘to make the long journey worth the while’. This was, however, not supported by the therapists, as they believed that the patients would then suffer from lack of energy that would compromise the outcome of one or the other activity. Both patients and the therapists reported that the patients were exhausted after each session, and this slowed down all movements notably.

The third theme describes key factors for success, and three categories explain the content. The category ‘Togetherness’ describes how one of the most important factors for success was the friendship that developed between the group members. The participants spontaneously gathered afterwards to have coffee and discuss Parkinson-related issues. According to the therapists, group cohesion was enhanced if the group contained some sociable and interacting persons. Moreover, they felt that a group of three or four members would probably not achieve the same positive group experiences.

The category ‘Leadership competencies’ highlights how the therapists were appreciated for being including, enthusiastic, and encouraging, although sometimes experienced as being a bit too brisk. From the therapists’ perspective, the Parkinsonian typical reduction of facial expressivity made it difficult to see if the patients enjoyed the training. This lack of response led to an urge to be even more enthusiastic and energetic, which afterwards made them feel exhausted: ‘It looks as if they don’t enjoy it at all with these masked facial expressions, but they keep returning, so they must think it’s worth it to come here (laughs)’. It was also appreciated that the therapists clearly made efforts in choosing familiar music and encouraged the participants to bring their favourite music.

The category ‘Contextual components’ includes discussions about environmental issues. Several things provoked irritation that were not part of the intervention, but rather the surrounding environment. It was reflected upon that there were far too few parking lots, and spending energy on searching for a parking lot was exhausting. Those who were dependent upon transportation service were discouraged by the lack of flexibility when ordering the taxi service, and the energy that this consumed also affected them negatively.

## Discussion

This study shows that the group-based music intervention may add value to psychological aspects such as mood, alertness, and quality of life, in patients with Parkinson’s disease. The study does not, however, support the efficacy of the therapy in producing immediate or lasting improvements in motor-cognitive dual-tasking, cognitive function, balance, or freezing of gait, when compared with the control group. Supplementary interviews revealed that small improvements were noted by patients and therapists in the intervention group, but none were reproduced in the functional tests. The results therefore suggest that the Ronnie Gardiner Method should not be adopted in clinical practice in preference to more robust evidence-based movement therapies,^1,4,5^ including dancing,^2^ in Parkinson’s disease.

There are several possible reasons to consider with respect to why the intervention was not effective. First, the focus was on stability and movement quality, and the challenge of the exercises may therefore have been too low to improve balance during dual-task conditions.^27^ Second, the intervention was not individualized, which meant that the group could only progress at the rate of the poorest responder in the group.^28^ This was frustrating to some patients, and the therapists found it difficult to negotiate the right level of challenge. A more personalized approach would most likely improve the conditions for training effects.^29^ Finally, the short-term improvement for quality of life and the reduction of concerns about falling may be due to the placebo effect, especially since no cognitive or motor outcomes were improved in the intervention group compared to the control group. It should also be considered that the study was underpowered and had a large amount of missing data, which makes the results less clear.

It should also be noted that 50% of the patients scored less than the normal cut-off (26/30)^26^ on the MoCA, and this is likely to have affected the ability to learn the complex dual-task movements for many participants, as well as to have contributed to the non-significant results.^30^ Research shows that patients with Parkinson’s disease may experience difficulties in learning new complex motor skills and therefore require longer time than healthy adults,^31^ and this was confirmed by the therapists in this study. Future studies should examine the results obtained for different sub-groups, although this will require a larger sample.

The reduction regarding concerns about falling is in line with other exercise intervention studies.^32^ There is, however, a risk that the reduction may in fact increase the risk of falls in the absence of improved mobility and balance. Whether the results are clinically meaningful in terms of fewer falls remains therefore to be investigated, but since 80% of the patients experienced poor balance at baseline these findings may be of relevance.

There has been a call for the development of activities that encourage social interaction to improve well-being in patients with Parkinson’s disease.^33^ The music-based group therapy under study includes sensory stimulation and music in pleasant social contexts, and thus has the potential to increase task enjoyment and improve the effectiveness of motor rehabilitation interventions.^31^ Patients from the intervention group were united in the perception that the activities were enjoyable (‘a winning concept’), which may be a contributing factor to becoming more cheerful and alert. This is in agreement with qualitative studies on stroke survivors.^34,35^ Having fun together as a group in a stimulating activity may indeed add value in terms of peer support, motivation through group accountability, and social interactions that may have effects above and beyond the intervention itself.^36^

The short-term improvements in self-perceived quality of life in the intervention group confirmed the positive trend of our previous feasibility study.^8^ Our findings are contrasting to a recently published systematic review and meta-analysis that found no evidence that music-based movement therapies (including dancing) led to quality of life improvements in patients with Parkinson’s disease.^1^ The intervention group did not reach the minimal clinically important difference for Parkinson Disease Questionnaire-39.^37^ Although mean changes in our study were minor, they may have important clinical implications for patients with Parkinson’s disease. In contrast, the worsening of the control group did exceed the minimal clinically important difference.^37^

This study has limitations. There is a risk for a type II error as the study was underpowered. We used a design with repeated measurements in time, which should somewhat reduce this risk.^38^ The significant amount of missing data further reduced the data availability and might have influenced the quality of the analysis. Not using the intention-to-treat analysis further limits the robustness of the results. This limits the generalizability of our findings.

We did not control statistically for patients’ level of medication and not re-assessing patients at the exact same time of the day. The level of dopaminergic medication differs between individuals, and the responsiveness of medication may also vary over the day due to differences in disease severity or time of onset.^39^ The fact that six patients from the intervention group – in contrast to one patient from the control group – decreased their Levodopa equivalent dosage during the study period is worth noting. Future studies are advised to include Levodopa equivalent dosage as an outcome measure and to control for levels of medication statistically.

The majority of measurements used are established as valid and reliable for patients with Parkinson’s disease, except for the Timed-Up-and-Go subtracting serial 7’s and the Cognitive Assessment Battery, which was developed for people with mild cognitive impairment.^18^ In addition, it may be questioned whether the reduction of the mean score by 3.2 points for the Falls Efficacy Scale International represents a real change rather than merely an expression of a measurement error.^40^ The primary outcome may not have been in line with the therapy, and a different test may have been more appropriate.

We found that mixing methods offered complementary insights. The qualitative findings added contextual information with the potential to provide a more complex understanding of the different dimensions of the music-based group therapy than quantitative measurements alone might provide. Contextual information may be valuable for optimizing treatment effects.^9^ Another strength is the addition of a complete audit trail including a discussion about reflexivity and risk of bias in the qualitative data (Supplementary file III).

With respect to clinical practice, this study does not support the efficacy of the Ronnie Gardiner Method in producing gains in dual-task ability, balance, cognition, or freezing of gait, in patients with Parkinson’s disease. There are, however, indications that the therapy was socially and psychologically beneficial. The therapy was appreciated for its playfulness, the use of music, and the engaging therapists. In addition, the therapy was safe to use, and the attendance rate was high. The group-based music intervention may therefore be useful in cases when motivation for physical exercise is low.

**Clinical messages**

- The group-based music intervention, the Ronnie Gardiner Method, did not improve dual-task ability, cognition, balance, or freezing of gait in patients with Parkinson’s disease.
- A short-term reduction for concerns about falling was found, but not for balance.
- Patient-reported outcomes and interviews indicate that the group-based therapy adds value to mood, alertness, and quality of life.

## Supplemental Material

Supplementary_file_III_audit_trail – Supplemental material for Group-based music intervention in Parkinson’s disease – findings from a mixed-methods study Click here for additional data file. Supplemental material, Supplementary_file_III_audit_trail for Group-based music intervention in Parkinson’s disease – findings from a mixed-methods study by Petra Pohl, Ewa Wressle, Fredrik Lundin, Paul Enthoven and Nil Dizdar in Clinical Rehabilitation

Supplementary_file_II_intervention – Supplemental material for Group-based music intervention in Parkinson’s disease – findings from a mixed-methods study Click here for additional data file. Supplemental material, Supplementary_file_II_intervention for Group-based music intervention in Parkinson’s disease – findings from a mixed-methods study by Petra Pohl, Ewa Wressle, Fredrik Lundin, Paul Enthoven and Nil Dizdar in Clinical Rehabilitation

Supplementary_file_I_CONSORT – Supplemental material for Group-based music intervention in Parkinson’s disease – findings from a mixed-methods study Click here for additional data file. Supplemental material, Supplementary_file_I_CONSORT for Group-based music intervention in Parkinson’s disease – findings from a mixed-methods study by Petra Pohl, Ewa Wressle, Fredrik Lundin, Paul Enthoven and Nil Dizdar in Clinical Rehabilitation
