# Supplementary material for: Group-based music intervention in Parkinson’s disease – findings from a mixed-methods study
Source: Clin Rehabil. 2020 Feb 19;34(4):533–44. doi: 10.1177/0269215520907669 (PMC7132435; doi:10.1177/0269215520907669)
Supplement: Supplementary_file_III_audit_trail – Supplemental material for Group-based music intervention in Parkinson’s disease – findings from a mixed-methods study [file Supplementary_file_III_audit_trail.pdf]

Audit trail for qualitative analysis

To increase transparency and to ensure dependability and confirmability, an audit trail is presented, outlining the theoretical framework underlying this study and the steps in the qualitative content analysis. A section of reflexivity is also included as recommended in the Consolidated criteria for reporting qualitative research (COREQ).[1] At the end of the document, there is an extended version of the qualitative results.

Theoretical framework
According to Lewin et al (2009), qualitative methods should be used alongside randomised controlled trials (RCT).[2] We therefore decided to add qualitative methods to the more positivistic quantitative approach. The philosophical position underlying the qualitative part of this study relied on an interpretivist orientation. The epistemological position of interpretivism is subjectivism, and the ontological position is relativism. Furthermore, an interactionist perspective was adopted as methodological approach, where people are actively shaping their culture and social roles through interaction and interplay with other individuals in social contexts.

Research team and reflexivity
Five researchers were involved in this study. The research team (i.e., the authors) was composed of an interdisciplinary team with PhD degrees and experiences from different methodological orientations, and this was expected to broaden the interpretation of the analysis. P.P. and P.E. are physiotherapists and assistant professors; E.W. is an occupational therapist and associate professor; F.L. and N.D. are movement disorders specialists; N.D. is associate professor. All researchers have previous experience from rehabilitation in different settings and from quantitative methodology. Three researchers (E.W., P.E., and P.P.) have formal training and several years of documented experience from qualitative methods.

P.P. is a non-practicing certified practitioner of the Ronnie Gardiner Method (RGM), and thus there is a high risk of bias that may raise questions about the trustworthiness, credibility and dependability of our qualitative findings. To reduce this risk, a number of precautionary measures were taken: no interviews were conducted by P.P.; the qualitative analyses were performed together with P.E. and E.W. who have no previous experience from, nor pre-understanding of, RGM; and the analysis stayed on a manifest level, i.e., a low degree of interpretation.[3] No other members of the research team had any connections with RGM. In addition, statistical analyses were performed by an independent statistician.

None of the two delivering physiotherapists (the instructors), nor the music therapist who performed the integrity check, were involved in any way in the study except for their role as therapists.

Data collection
Focus groups and individual interviews were used for data collection, as suggested by Lambert.[4] The focus group discussions gave rich data, because the participants in focus groups are known to interact with each other, inspiring to new thoughts. The focus group technique is built on the notion that the group interaction encourages study participants to explore and clarify individual as well as shared perspectives. Focus groups are typically used to explore views on health issues, programs, interventions and research.[1] We did, however, also want to capture individual experiences that would perhaps not be aired in larger groups, and therefore added a number of individual interviews.

Focus groups were conducted within one week after the intervention. To reduce the risk of selection bias, all participants were invited to take part in semi-structured focus groups. One focus group was also conducted with the two delivering physiotherapists. All focus groups were facilitated by EW. Additional individual interviews were conducted with eight purposively selected patients (6 men and 2 women) with a variety of ages, gender, and disease duration in order to extract information based on individual experiences. These interviews were conducted by four physiotherapy students supervised by PE. The location of the individual interviews was the participant's own housing. There were no

known previous personal relationships between the patients and the interviewers. The characteristics of all participants from the interviews are presented in Table 1.

Table 1. Participant characteristics for focus groups, and number of those who also took part in individual interviews.

Focus Groups	Age, years, mean (range)	Years with disease mean (range)	Male, n (%)	Individual interview
FG 1 (*n* = 4)	73 (65 – 81)	3.3 (2 to 4)	3 (75)	Two
FG 2 (*n* = 4)	65 (61 – 70)	9.3 (6 to 17)	3 (75)	Three
FG 3 (*n* = 4)	72 (53 – 80)	3.5 (2 to 6)	2 (50)	--
FG 4 (*n* = 5)	71 (66 – 76)	5.4 (1 to 11)	4 (80)	Three
FG 5 (*n* = 4)	74 (69 – 78)	7 (6 to 9)	2 (50)	--
FG 6 (*n* = 4)	66 (57 – 72)	9.8 (2 to 16)	3 (75)	--
FG 7 (*n* = 2)	NA	NA	0 (0)	--

NA: Not applicable

A semi-structured interview schedule was used for all interviews (Box 1). The schedule was piloted in a practice interview and discussed with PE and PP. Since no subsequent adjustments were made, it was included in the analysis. The questions to the delivering physiotherapists were similar but slightly adapted. Prompts such as *"In what way was it so? Can you elaborate what you mean?"* complemented the schedule. Digital recordings of the interviews were transcribed verbatim (focus groups by E.W.; and face-to-face interviews by the students; all were checked for accuracy by P.P. with cross-reference to the audio files) and anonymised by a unique number prior to the analysis.

Box 1. Semi-structured Interview Guide for Focus Groups and Individual Interviews

- Describe in your own words how you experienced training with the Ronnie Gardiner Method (training contents, leadership, peers, environmental conditions, etc).
- Did you notice any barriers / facilitating factors to keep up with the movements? If so, which ones?
- Did you experience any changes over time (physical, social, mood, dual-task ability)? If so, which ones?
- Did the training have any impact on your everyday life? If so, in what way?
- Do you have any thoughts about the structure and the contents of the sessions or the Method? Suggestions for modifications to optimise the training effects?

Qualitative content analysis

Data were analysed thematically using qualitative content analysis[3] in order to identify differences and similarities of the contents. The process of analysing the material and deriving themes was done in a stepwise process between March and September 2017 and completed in two phases. First, focus groups were analysed by P.P. and E.W. with an inductive approach, i.e., derived from the data,[5] leading to main themes. Second, the face-to-face interviews were analysed by P.E. and P.P., but instead with a deductive approach with the pre-defined themes as starting point.

In the first phase, the anonymised transcripts of the focus groups were read repeatedly and independently by P.P. and E.W. to start the process of identifying meaning units and to acquire a good grasp of the whole. Meaning units were comprised of units of words or sentences that corresponded solely to the research question. The meaning units were compared until agreement was reached and were then condensed into labelling codes by both authors together (Table 2). Codes were close to the text (manifest level) and described the content of the meaning unit with a few words without losing the core content of the unit. The codes were grouped into categories with similar utterances under a descriptive concept. Further, the categories were merged into themes, labelled with a description of their contents. Final themes were agreed upon by all authors.

Table 2. Examples of the analysis process with meaning units, codes, categories and themes.

Meaning unit	Codes	Category	Theme
"You needed to remain focused and concentrated. As soon as you start thinking about something else you are completely lost." [FG 2]	Need to stay focused<br><br>Immediately lost when concentration breaks	The therapy itself	Perspectives on treatment contents
"The question is whether they should use easier or more complicated exercises. It feels as if the easy exercises are for fun, but the more difficult one's are for brain training." [FG 5]	Complicated exercises train the brain<br><br>Easy exercises are for fun	Design of treatment sessions	Perspectives on treatment contents
"I think the break is perfect, you can just sit and relax. "<br>"I agree. And I believe that prolonging the therapy with half an hour would probably be too exhausting."<br>"Yes, I think it is important to feel fresh when you leave." [FG 3]	Perfect with a break<br><br>Probably exhausting with 1,5 hours<br><br>Important to keep energy afterwards	Design of treatment sessions	Perspectives on treatment contents
"I think the language is strange. Perhaps it would be easier to learn if we had more homework."<br>"Yes, this could have encouraged us even more to learn the words." [FG 6]	Strange words [RGM specific]<br><br>Homework could encourage learning words	Design of treatment sessions	Perspectives on treatment contents

The face-to-face interviews were then analysed with the same technique by P.P. and P.E. together and with the use of constant comparison to synthesise the data. To increase credibility and trustworthiness, direct quotes from different interviews (focus groups and individual) are provided to support the findings.

RESULTS
This is a more extensive version of the results from the main manuscript.

Theme 1: Expectations vs. Results
Anticipations
Most patients were excited to try something new and expected the music to engage them. The physiotherapists had prepared the sessions thoroughly in order to make it a positive experience for everyone. They anticipated that the patients would get more out of it if the training was experienced as enjoyable.

Experienced Effects
Generally, the training was perceived as energising; it 'kicked off the ignition', or the brain 'perked up'. Patients described feelings of improved posture and dexterity with less tremor, a smoother body, higher energy levels with improved ability to concentrate, improved sleep quality, and being more cheerful: *"It sounded a bit like humbug that it could affect the brain, but it felt really good! I honestly believe this is helping, I don't think it's imagination."* (Agreement from others) (Group 4) These effects had a positive impact on daily life. For example, patients experienced improvements in staying focused while driving, or while solving crossword puzzles. Lack of training effects were also reflected upon: *"The tests will ultimately reveal whether there were any effects."* (Group 2)

The physiotherapists also noted subtle changes among the patients, especially improved mood and energy levels, movement quality, and a better ability to stay focused throughout an entire piece of music. Another example was timing; at times they deliberately placed empty spaces within the note systems (i.e., rests of one beat), and the ability to *not* move correctly was improved. This was confirmed by an individually interviewed man: *"Sometimes there is a section when you do nothing, there is a hole, and you have to stop yourself from clapping or doing something else. This ability has definitely improved."* One interfering factor according to the physiotherapists was that the patients' symptoms fluctuated, which made it more difficult for the physiotherapists to observe effects.

Theme 2: Perspectives on Treatment Contents

The Method Itself

Patients agreed that the concept with the red and blue symbols in the shape of hands and feet within note systems was something out of the ordinary, although the 'nonsense-words' were hard to learn. The training was not experienced as heavy or sweat inducing, but instead easy-going, positive, and fun: *"It's a winning concept! It's a combination of the programme itself, the rhythm of the music, the fellowship, and the enthusiastic leaders." "Yes, the programme is so much fun that you become exhilarated." "I agree, there is a lot of physical activity for one hour, and this gives you endorphins, and that is the 'joy substance' of the body." "Yes, and there is much memory training."* (Group 5)

To read the note systems row by row while simultaneously coordinating arms and legs and say the correct words out loud to the beat of music was a real challenge. When the concentration was broken, one was instantly lost. The physiotherapists observed that usually movements were prioritised, while the words were lost, or that the movements were poorly performed while uttering the words. The patients needed much visual support, i.e., the therapist continuously pointed at the note system beat by beat. Once the pointing stopped the patients were often lost. This pointing had another downside; while turning the back on the patients, the physiotherapists were unable to observe the participants for safety reasons. Therefore, it was experienced as advantageous to have two instructors.

Design of Treatment Sessions

The focus groups perceived the exercise regime as well-planned and well-structured. The patients agreed that twice weekly one-hour sessions were just right, and this opinion was shared by the physiotherapists who thought that once a week would limit motor learning. Many of the exercises were performed sitting down by choice, even when the physiotherapists gave the option to stand up. One man from the face-to-face interviews said: *"I preferred to stand up while doing the exercises, it's a bit like dancing, at least sway a little (laughing). If you get it to work, it's more fun than to just sit down and 'boom', 'chic', and so on. Music affects you very much physically!"*

The physiotherapists prioritised movement quality: "If they are unstable, it's better if they sit down and do the movements properly; otherwise they will fail to perform them correctly." The difficulty of the exercises was also reflected upon. The physiotherapists observed that many patients had great difficulties in learning the complex motor-cognitive skills but found it difficult to negotiate the right level to fit everyone, because the group was heterogeneous. Instead they tried to compromise, but this had consequences for the patients who were quick learners: *"I had expected the training to be much harder and more challenging, I wanted to challenge my abilities more than it did. Only at the end they increased the speed and it became more challenging to me."* (man, individual interviews) For this patient the exercises soon became repetitive.

There were conflicting ideas about how to improve the sessions. Patients suggested adding rotational movements to the exercises and to skip the initial facial gymnastics. According to them, more homework was needed to enhance motor learning, but the physiotherapists disagreed; in their expe-

rience, homework was rarely carried out. Another suggestion made by the patients was to combine RGM with other physiotherapy specific activities, such as taking a walk together, doing gymnastics, or adding 15 minutes of resistance training before or after the sessions, because *"this is not enough as physical exercise"*, and *"to make the long journey worth the while"*. This was, however, not supported by the physiotherapists, as they believed that the patients would then suffer from lack of energy that would compromise the outcome of one or the other activity. Both patients and the physiotherapists reported that the patients were exhausted after each session, and this slowed down all movements notably.

Theme 3: Key Factors for Success

Togetherness

One important factor for success was the sense of belonging. Friendships developed between the group members, and it was perceived as crucial to meet (new) peers, and to have fun together. The participants spontaneously gathered afterwards to have coffee together, and they found this to be a good opportunity to share Parkinson specific information. According to the physiotherapists, *"the individuals shape the group"*; they thought group cohesion was enhanced if the group contained some sociable and interacting persons, and if the patients contributed with their favourite music, which led to interesting discussions. Moreover, they felt that a group of 3 or 4 members would probably not achieve the same positive group experiences, and that a small group may be more sensitive to making mistakes, because no one wants to stand out as a failure: *"It's almost like singing in a choir, nobody cares if you miss the odd tone, but if you are singing solo everyone will hear it."* (Physiotherapist)

Leadership Competencies

The therapists were appreciated for being including, skilled, enthusiastic, encouraging, and playful, although sometimes experienced as being a bit too brisk. From the physiotherapists' perspective, the Parkinsonian typical reduction of facial expressivity, combined with hypokinesia, made it difficult to see if the patients enjoyed the training. This lack of response led to an urge to be even more enthusiastic and energetic, which afterwards made them feel exhausted: *"It looks as if they don't enjoy it at all with these masked facial expressions, but they keep returning, so they must think it's worth it to come here (laughs)."* It was also appreciated that the therapists clearly made efforts in choosing familiar and rhythmical music, and that they constantly encouraged the participants to bring their favourite music to the sessions.

Contextual Components

The location of the intervention was a light and spacious neurological rehabilitation centre, but several things provoked irritation that were not part of the intervention, but rather the surrounding environment. It was reflected upon that there were far too few parking lots. Due to the disease, the patients had a limited amount of energy, and spending it on searching for a parking lot was exhausting. Those who were dependent upon transportation service were discouraged by the lack of flexibility when ordering the taxi service, and the energy that this consumed also affected them negatively.

REFERENCES

1. Tong A, Sainsbury P, Craig J. Consolidated criteria for reporting qualitative research (COREQ): a 32-item checklist for interviews and focus groups. *Int J Qual Health Care.* 2007;19(6):349-357.
2. Lewin S, Glenton C, Oxman AD. Use of qualitative methods alongside randomised controlled trials of complex healthcare interventions: methodological study. *BMJ.* 2009;339:b3496.
3. Graneheim UH, Lundman B. Qualitative content analysis in nursing research: concepts, procedures and measures to achieve trustworthiness. *Nurse Educ Today.* 2004;24(2):105-112.
4. Lambert SD, Loiselle CG. Combining individual interviews and focus groups to enhance data richness. *J Adv Nurs.* 2008;62(2):228-237.
5. Green J, Thorogood N. *Qualitative methods for health research.* 3rd ed. Los Angeles: SAGE; 2014.
