# Supplementary material for: Group-based music intervention in Parkinson’s disease – findings from a mixed-methods study
Source: Clin Rehabil. 2020 Feb 19;34(4):533–44. doi: 10.1177/0269215520907669 (PMC7132435; doi:10.1177/0269215520907669)
Supplement: Supplementary_file_II_intervention – Supplemental material for Group-based music intervention in Parkinson’s disease – findings from a mixed-methods study [file Supplementary_file_II_intervention.pdf]

Supplementary file II. Checklist for Reporting Music-based Interventions (Robb et al, 2011)

Music-based Intervention Reporting Criteria

This checklist is based on the recommendations by Robb *et al* (2011),[1] in order to increase clarity of music-based interventions in a more structured way, as requested in the Consolidating Standards for Reporting Clinical Trials (CONSORT), but has been further extended.

A: Intervention Theory
Music-based interventions have in general been found beneficial to people with Parkinson's disease (PD).[2,3] Music leads to immediate effects by activating action-related processes, as well as long-term effects such as improved gait in people with PD.[4] The most potent component is *rhythm*, because it acts as an external cue, bypassing the damaged basal ganglia.[4] The Ronnie Gardiner Method (RGM) is a music-based intervention based on auditory rhythm (i.e., beat-based music perceived through hearing) with the goal to improve postural control, enhance gait, and improve cognitive function such as memory. Auditory rhythm given through music influences the kinetic system through synchronisation and adjustments of muscles to the stimuli of music and facilitates movement synchronisation, coordination, and regularisation.[5] The exercises can be performed either sitting down or standing up. While standing, many weight-shifts are involved, challenging anticipatory and reactive postural control. By means of the choice of the symbols and their order, exercises are created to practice starting and stopping, walking forwards and backwards, or stepping aside.

With respect to dual-task performance, RGM is a motor-cognitive intervention that incorporates multiple external cues. Apart from the auditory cues, somatosensory cues through body percussion (e.g., handclaps, stomping with feet, slapping thighs) and visual cues (projected symbols on a screen) are also used.[4] These proposed effect mechanisms are expected to improve motor control and cognitive skills such as concentration, information processing, working memory, and thereby dual-task performance. For information, please visit the official website www.ronniegardinermethod.com.

B: Intervention Content
Each session was initiated with soft stretching movements and breathing exercises, followed by 50 minutes of exercises typical the RGM,[4] and ended with soft classical music. Short breaks were provided between the exercises.

B.1: Person Selecting the Music
The music was pre-selected each week by the practitioners based on: a) appropriate tempo (defined by beats per minute, BPM) for the participants; b) different time signatures (mainly 4/4); and c) participants' selections. Efforts were made to choose familiar "feel-good" music, or uplifting music to awaken memories from the participants' adolescence with the potential to influence the atmosphere, mood, and the impact of the exercises in a positive way. Participants were also encouraged to bring favourite music from home or were given opportunities to request any piece of favourite music to optimise involvement and enjoyment.

B.2: Music
Published music was continuously used. The qualities of the music (e.g., form, melody, harmony, voicing, and tonality) were not considered, as the focus was on using rhythmical music with a steady beat to the exercises. Examples are given below:

**Title:**	**Artist:**	**Tempo:**
Happy together	The Turtles	61 BPM
Say you, say me	Lionel Ritchie	64
Get up, stand up	Peter Tosh	69
Lonely avenue	Ray Charles	72
Layla	Eric Clapton	94
The French march	The Chieftains	108
Wade in the water	Eva Cassidy	112
Billy Jean	Michael Jackson	116

During short breaks and at the end of each session, slow and soft music was used to elicit a relaxed atmosphere, with the addition of a projected picture of nature on the screen to rest the eyes upon.

B.3: Music Delivery Method
Recorded music was used, delivered through speakers placed at one side of the room. This allowed participants to simultaneously attend to the music, the instructions, and the other group members, eliciting attention. The volume was controlled by the interventionists and set based on the participants being able to clearly hear the beat of the music, as well as the instructions. The intention was also to rise physiological arousal levels, adding to the invigorating effect. The volume was immediately lowered upon request if any of the participants expressed discomfort with respect to auditory health and unhealthy sound levels. No decibel level was noted.

B.4: Intervention Materials
No musical instruments or items to create musical sounds were used. Non-musical materials were: audio playback equipment (Onkyo Compact disc player DX-7355), a laptop (HP Elite Book) with output VGA and electrical source, a white cloth, and a connected projector (Logitech). A remote (battery powered) was used to change the slides of the PowerPoint presentation with the note systems.

B.5: Intervention Strategies
Certain note systems – referred to as *choreoscores* – with, for RGM unique, red and blue symbols resembling hands and feet were projected on a screen[a]. These symbols work as perceptual cues (colours and shapes). Each symbol is associated with a movement (e.g., stomp with right foot on the floor) and a certain verbal code (e.g., BOOM or CHIC). In this study, 13 out of the existing 19 RGM specific symbols were used. To the rhythm of music, the participants visually read the choreoscores row by row from left to right, while synchronising their motor rhythm with the auditory rhythm and pronouncing the verbal codes loudly beat by beat. Every symbol has its own movement and verbal code to be pronounced while simultaneously performing the movement. When new symbols were introduced, the same pedagogical model was used in order to facilitate learning: first the word was spoken out loud repeatedly, then the motor skills were practiced repeatedly, and last all elements were put together, also repeatedly. Finally, the new symbol was practiced to music at an appropriate tempo. Participants occasionally received handouts with encouragement to repeat the movements at home to facilitate learning.

The exercises were performed either sitting down or standing up. If the movements were experienced as being too challenging, the participants were offered a stationary parallel bar to hold on to for safety. For progression, the note systems were delivered with increasing musical tempo, and/or with more complex symbols and movements.

C: Intervention Delivery Schedule
Sessions were delivered twice weekly (Mondays and Fridays) for twelve weeks (n= 24). Each session was 60 minutes. Participants were encouraged to practice at home, but without control.

D: Interventionists
To strengthen arguments for an intervention effect rather than a person effect, two experienced physiotherapists provided the intervention, both with a bachelor’s degree and certified RGM practitioners. The certification process typically involves completing three 2-day courses with Mr. Ronnie Gardiner himself or teachers appointed by Mr. Gardiner. Both practitioners had several years of practice from teaching RGM (4 and 10 years, respectively) and of working with people with PD, individually as well as in groups (27 and 19 years). The interventionists both had much experience of

[a] Due to publication rights, details of these symbols/movements/codes cannot be described in detail here. However, examples can be found on Google or YouTube when searching for “Ronnie Gardiner Method”. Any certified RGM practitioner are welcome to contact the first author (principal investigator) and take part of all note systems used in the intervention on request in order to replicate the intervention.

creating choreoscores suitable for different neurological diseases and age groups. The principal investigator offered meetings regularly to discuss any possible difficulties with the programme.

E: Fidelity Strategies for Treatment Delivery
A third qualified RGM practitioner (music therapist) was contracted to check for accuracy, i.e., that the protocol was delivered as intended. This was done at the 11th session.

F: Setting
The intervention was delivered in a spacious room at a neurological rehabilitation center in Linköping, Sweden. A stationary parallel bar was placed at one side of the room for those who needed more support during stance.

G: Unit of Delivery
The intervention was delivered in groups of individuals for the potential benefit of social interaction, peer support, and motivation through group accountability. Because of the size of the room, a pre-defined maximum amount of 15 people in the group was decided upon.

References

1. Robb SL, Burns DS, Carpenter JS. Reporting guidelines for music-based interventions. *J Health Psychol.* 2011;16(2):342-352.
2. Zhang S, Liu D, Ye D, Li H, Chen F. Can music-based movement therapy improve motor dysfunction in patients with Parkinson's disease? Systematic review and meta-analysis. *Neurol Sci.* 2017;38(9):1629-1636.
3. de Dreu MJ, van der Wilk AS, Poppe E, Kwakkel G, van Wegen EE. Rehabilitation, exercise therapy and music in patients with Parkinson's disease: a meta-analysis of the effects of music-based movement therapy on walking ability, balance and quality of life. *Parkinsonism Relat Disord.* 2012;18 Suppl 1:S114-119.
4. Pohl P. The Ronnie Gardiner Method: An innovative music-based intervention in neurorehabilitation with focus on Parkinson's disease. *Neurophysio and Rehab.* 2018(1):32-37.
5. Raglio A. Music Therapy Interventions in Parkinson's Disease: The State-of-the-Art. *Front Neurol.* 2015;6:185.
